# Safety of direct-acting oral anticoagulant (DOAC) prescribing: OpenSAFELY-TPP analysis of 20.5 million adults’ electronic health records

**DOI:** 10.3399/BJGPO.2023.0163

**Published:** 2024-07-29

**Authors:** Karen Homan, Rachel Seeley, Louis Fisher, Sajida Khatri, Katie Smith, Tony Jamieson, Victoria Speed, Carol A Roberts, Amir Mehrkar, Sebastian Bacon, Brian MacKenna, Ben Goldacre

**Affiliations:** 1 PrescQIPP C.I.C, London, United Kingdom; 2 Medicines Safety Improvement Programme, NHS England, London, United Kingdom; 3 The Bennett Institute for Applied Data Science, Nuffield Department of Primary Care Health Sciences, University of Oxford, Oxford, United Kingdom

**Keywords:** DOAC, creatinine clearance, COVID-19, patient safety, electronic health records

## Abstract

**Background:**

During the COVID-19 pandemic many patients were switched from warfarin to direct-acting oral anticoagulants (DOACs), which require the creatinine clearance (CrCl) calculated to ensure the correct dose is prescribed to avoid bleeding or reduced efficacy.

**Aim:**

To identify the study population proportion prescribed a DOAC. Of these, the proportion with recorded: weight, estimated glomerular filtration rate (eGFR), creatinine, CrCl and atrial fibrillation (AF). To analyse the proportion of patients with recorded AF and CrCl prescribed a recommended DOAC dose.

**Design & setting:**

A retrospective cohort study of 20.5 million adult NHS patients’ electronic health records (EHRs) in England in the OpenSAFELY-TPP platform (January 2018–February 2023).

**Method:**

Patients on DOACs were analysed for age, sex, recorded weight, eGFR, creatinine, CrCl and AF. Prescribed DOAC doses in patients with recorded AF were compared with recommended doses for recorded CrCl and determined as either recommended, higher than recommended (overdose), or lower than recommended (underdose).

**Results:**

In February 2023, weight, eGFR, creatinine, CrCl, and AF were recorded in 72.8%, 92.4%, 94.3%, 73.5%, and 73.9% of study population, respectively. Both AF and CrCl were recorded for 56.7% of patients. Of these, 86.2% received the recommended, and 13.8% non-recommended, DOAC doses.

**Conclusion:**

CrCl is not recorded for a substantial number of patients on DOACs. We recommend that national organisations tasked with safety, collectively update guidance on the appropriate weight to use in the Cockcroft–Gault equation, clarify that CrCl is not equivalent to eGFR, and work with GP clinical system suppliers to standardise the calculation of CrCl in the EHR.

## Introduction

During the COVID-19 pandemic many patients were switched from warfarin to a direct-acting oral anticoagulant (DOAC): apixaban, edoxaban, dabigatran, or rivaroxaban. This switch occurred to continue to keep patients on anticoagulants as safe as possible and reduce the impact on service provision to take on international normalised ratio (INR) monitoring in the community for the large number of patients who were housebound through COVID-19 infection, self-isolating, and adopting social distancing strategies.^1^ Compared with warfarin, DOACs require less frequent monitoring, have a faster onset of action, predictable pharmacokinetics, and fewer food and drug interactions.^2^ Although DOACs have these advantages, their use increases the risk of bleeding and can cause serious, potentially fatal, bleeds. The Medicines Healthcare products Regulatory Agency (MHRA) warns that DOACs should be used with caution in patients at increased risk of bleeding such as in older patients, those with low body weight, or renal impairment.^3^ In England renal function is commonly measured by estimated glomerular filtration rate (eGFR).^4^ However, it is recommended that when prescribing and monitoring DOACs, renal function is assessed by calculating the CrCl using the Cockcroft–Gault method, as eGFR can overestimate the renal function and increase the risk of bleeding.^4–6^ People with atrial fibrillation (AF) are prescribed DOACs long term and so have a greater exposure time to DOACs compared with use in other indications.^4^

National clinical guidance issued during the COVID-19 pandemic suggested a process for switching from warfarin to a DOAC, which included checking the GP clinical system for urea and electrolytes (U&Es), recording weight, calculating CrCl, and ensuring that the DOAC is prescribed at an appropriate dose.^1^

The OpenSAFELY-TPP platform is a secure, transparent, open-source software platform, which allows for the analysis of 24 million NHS electronic health records (EHRs), covering 40% of general practices in England. OpenSAFELY has been used to deliver urgent academic and operational NHS service research on the direct and indirect impacts of the pandemic, including the changing pattern of anticoagulant use during the COVID-19 pandemic.^7^ It has also supported a national patient safety alert with the potentially inappropriate use of DOACs in people with mechanical heart valves.^8^

With the approval of NHS England, we set out to use the OpenSAFELY-TPP platform to describe the change in DOAC prescribing since the COVID-19 pandemic. We wanted to find out the proportion of patients prescribed a DOAC who had recorded weight, creatinine, eGFR, and CrCl in line with recommendations on calculating DOAC doses in individuals.^4–6^

## Method

### Study design and population

We conducted a retrospective cohort study, using the patient population within the OpenSAFELY-TPP platform, between January 2018 and February 2023. The study population included all patients each month who were: registered with a TPP practice; aged between 18 years and 120 years; who had not died before the start of the month and were prescribed a DOAC (rivaroxaban, apixaban, edoxaban, or dabigatran). Patient characteristics, including sex and indication for anticoagulation, were extracted for the whole study population. Weight (recorded, not recorded,<50 kg,>120 kg), creatinine, eGFR, CrCl (recorded, not recorded,<15 ml/min), AF, AF and CrCl, and the dose of DOAC were extracted, if coded in the EHR, from the study population between March 2022 and February 2023 for these analyses.

The proportion of patients with weight, eGFR, creatinine, and CrCl recorded were reported as a percentage and compared over the study period. For patients with recorded AF prescribed a DOAC and with a CrCl recorded, the actual DOAC dose was determined as either a recommended, higher than recommended (overdose) or lower than recommended (underdose) from the recorded CrCl.^1^ (Codelists used to ascertain patients prescribed DOACs are listed in Table 1.)

### Statistical analysis

Patient characteristics were summarised using descriptive statistics. The study measures are presented as percentages, including the proportion of the population prescribed a DOAC (all indications); percentages were reported monthly from January 2018–February 2023. The proportion of the population with recorded AF with each monitoring parameter measured within the past 12 months (weight, creatinine, eGFR, and CrCl), and the proportion of the population with a recommended (matched), overdose, or underdose DOAC dose, according to CrCl in patients with recorded AF, are also presented; percentages were reported monthly from March 2022–February 2023. The percentage change in each monitoring parameter measured was computed between July 2022 and February 2023, the latest data available.

### Data source

All data were linked, stored, and analysed securely within the OpenSAFELY-TPP platform, https://opensafely.org/, containing pseudonymised data on approximately 40% of the English population, including coded diagnoses, medications, and physiological parameters. No free-text data are included. Detailed pseudonymised patient data is potentially re-identifiable and therefore not shared.

### Software and reproducibility

We conducted data management and analysis using the OpenSAFELY software libraries, Python 3. All code for the OpenSAFELY platform is freely available under open licences for review and reuse on GitHub (https://github.com/opensafely). All code for data management and analysis for this paper is freely available under open licences for review and reuse on GitHub (https://github.com/opensafely/doacs-covid19).

### Patient and public involvement

OpenSAFELY has a publicly available website https://opensafely.org/ through which any patients or members of the public are invited to correspond to regarding this study or the broader OpenSAFELY project.

## Results

### Patient characteristics

Data were extracted for 422 539 and 430 778 adults in July 2022 and February 2023, respectively, meeting the study inclusion criteria. Table 2 provides the patient characteristics of the study population, clinical parameters recorded in the EHR, and DOAC dose analysis in July 2022 and February 2023.

Supplementary Table S1 provides, for each individual DOAC, the patient characteristics of the study population, clinical parameters recorded in the EHR, and DOAC dose analysis in July 2022 and February 2023.

### Population trends in DOAC prescribing

**Figure 1:**
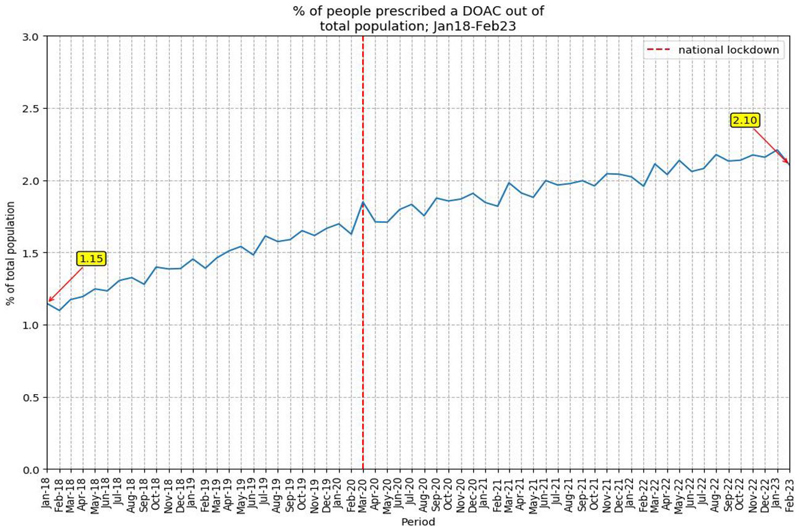
Percentage of people prescribed a DOAC out of study population (January 2018–February 2023). DOAC = direct-acting oral anticoagulant

DOAC prescribing increased from 1.15% to 2.10%, an increase of 82.6%, in the study population from January 2018–February 2023 (Figure 1).

The age groups with the largest number of people on a DOAC were the 70–79-years and >80-years age bands (Supplementary Figure S1). In February 2023, 44.5% of people prescribed a DOAC were female compared with 55.3% males (Supplementary Figure S2).

### Weight, eGFR, creatinine, CrCl, AF, AF and CrCl recorded

In February 2023, weight, eGFR, creatinine, CrCl, and AF were recorded in 72.8%, 92.4%, 94.3%, 73.5%, and 73.9% of study population, respectively. Table 3 illustrates the percentage change in the recording of weight, eGFR, creatinine, CrCl, AF, AF and CrCl in the past 12 months, which occurred in July 2022 and February 2023, the latest 8 months data available. The largest increases between July 2022 and February 2023 were seen for CrCl recorded (+42.0%) and AF and CrCl recorded (+15.4%). Decreases were seen for patients with a creatinine level but no CrCl recorded (–17.2%).

### Prescribed DOAC dosage in AF compared with recommended dose for patient’s CrCl

**Figure 2:**
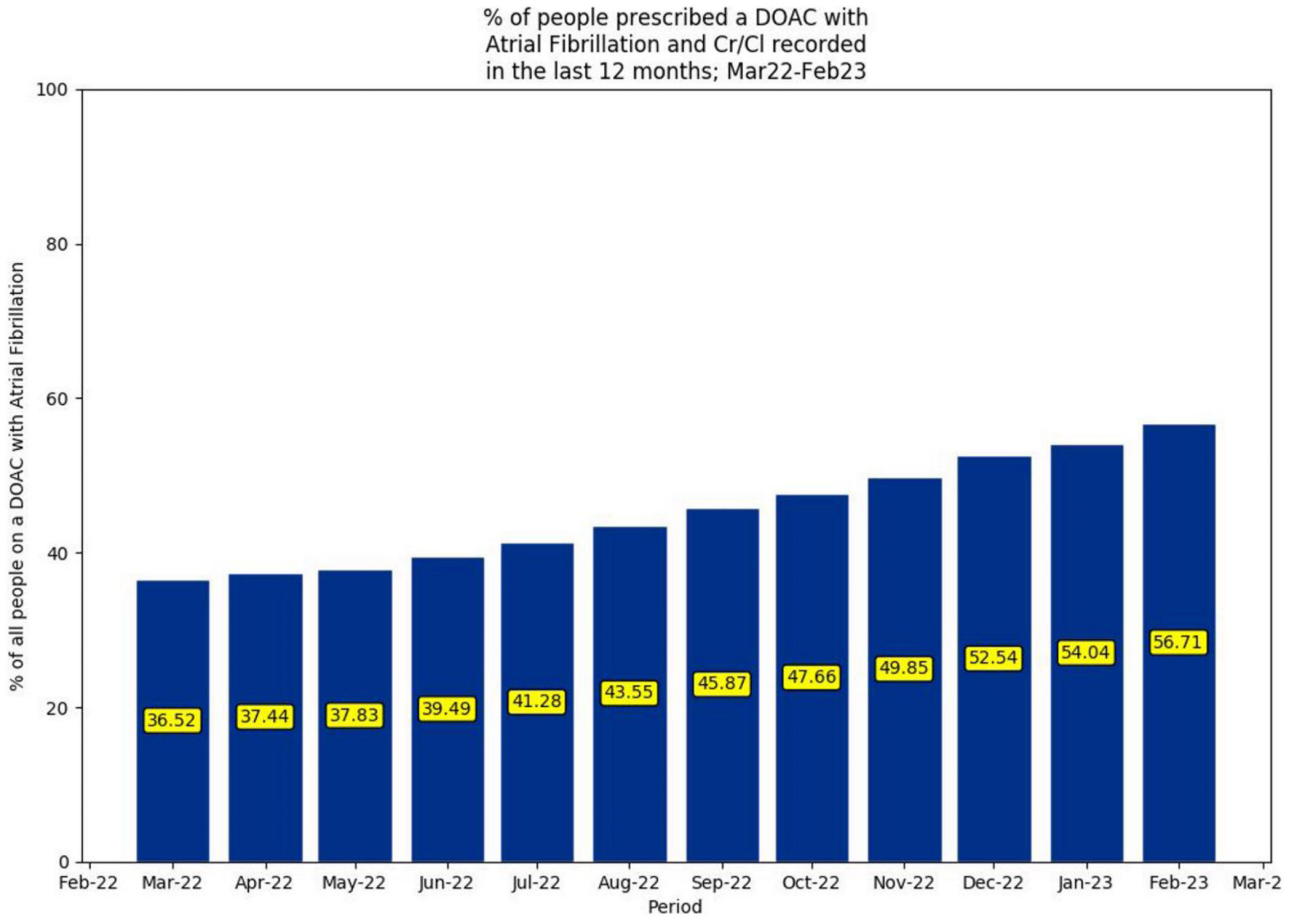
Percentage of people prescribed a DOAC with AF and CrCl recorded in the past 12 months (March 2022–February 2023). AF = atrial fibrillation. CrCl = creatinine clearance. DOAC = direct-acting oral anticoagulant

Between March 2022 and February 2023, 74.4%–73.9% of the study population prescribed a DOAC had a recorded diagnosis of AF. Figure 2 illustrates an increase from 36.5% to 56.7% in recorded CrCl in people prescribed a DOAC with recorded AF from March 2022 to February 2023.

**Figure 3:**
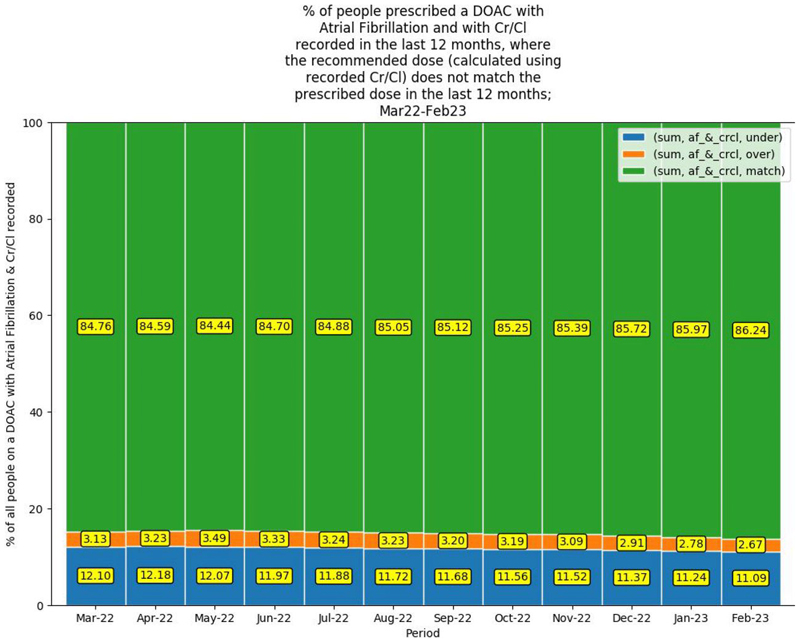
Percentage of people prescribed a DOAC with AF and with CrCl recorded in the past 12 months where the recommended dose (calculated using recorded CrCl) does not match the prescribed dose in the past 12 months; March 2022–February 2023. AF = atrial fibrillation. CrCl = creatinine clearance. DOAC = direct-acting oral anticoagulant. sum = total number of patients in which the AF and CrCl were recorded and the DOAC dose prescribed either matched, was an underdose or was an overdose compared to the DOAC dose calculated for their CrCl.

Figure 3 illustrates an increase in the percentage of patients, from 84.8%–86.2%, where the recommended DOAC dose (calculated using the CrCl recorded within the past 12 months) matched the prescribed dose (match); 13.8% received a non-recommended DOAC dose according to CrCl (2.7% higher than recommended dose [overdose]; 11.1% a lower than recommended dose [underdose]).

## Discussion

### Summary

DOAC usage since the COVID-19 pandemic was analysed in this large-scale study of approximately 20.5 million adult EHRs, covering 40% of general practices in England. The OpenSAFELY-TPP platform enabled the extraction of both recorded AF diagnosis and CrCl to determine the appropriateness of prescribed DOAC doses. There was an increase in DOAC use since the COVID-19 pandemic, with increases seen more in males compared with females and in older age groups. Encouragingly, 72.8%, 94.3%, 73.5% of patients had weight, creatinine, and CrCl, respectively, recorded in the past 12 months and the recording of CrCl increased substantially over this time in patients with AF and CrCl recorded. However, there remains a substantial proportion of people with recorded AF who do not have a CrCl recorded within the previous 12 months. While most people with a recorded AF were prescribed a recommended DOAC dose, 2.7% received a higher than recommended dose (overdose) and 11.1% a lower than recommended DOAC dose (underdose), according to CrCl.

### Strengths and limitations

The main strengths of this study are the scale, timeliness, and completeness of the underlying data. The OpenSAFELY platform runs analyses across the full, raw, single-event-level medical records of all patients at 40% of all GP practices in England, including all tests, treatments, diagnostic events, and other salient clinical and demographic information that has been coded and recorded. Using the OpenSAFELY platform, we were able to link a prescription for a DOAC, with recorded AF and CrCl. This presents an opportunity to investigate the safety and efficacy of DOACs prescribed in the cohorts where there is uncertainty or less published data; for example, weight <50 kg or >120 kg, CrCl <15 ml/min.

We also recognise some limitations. Only data that is coded in the EHR is included in the analysis and so any free-text data were not included. This could mean that our analysis has under-reported on parameters entered as free text rather than coded. Reduced face-to-face consultations may have limited actual body weights being recorded and documented. It is possible that some of the recommended DOAC doses calculated were inappropriate for patients with a dual diagnosis of venous thromboembolism (VTE) and AF, which require different DOAC dosing. In these patients, the VTE DOAC dose would be used in preference to the AF dose and therefore dose appropriateness could have been misclassified. Additionally, the appropriate DOAC dosing was calculated using only a patient’s recently recorded CrCl. Some DOACs, such as edoxaban, have additional criteria that contribute to the dosing recommendation, such as accounting for weight and concurrent treatment with interacting medications, which may mean that some doses have been misclassified.^9^

### Comparison with existing literature

Prescribing data show that between January 2018 and February 2023, DOAC prescribing increased from 0.82% to 1.58% of the total English population.^10^ This is in line with the findings in this analysis. A previous OpenSAFELY study also found an increase in DOAC prescribing at the outset of the COVID-19 pandemic owing to increased switching of anticoagulants from warfarin to DOACs.^7^

Our analysis, which found that 13.8% of people with recorded AF on a DOAC have been prescribed a non-recommended dose for their recorded calculated CrCl, is in line with Khachatryan *et al* who found that 18.0% of patients prescribed a DOAC received an incorrect dose from data up to March 2021.^11^ Our analysis has shown a 4.2% decrease in non-recommended DOAC doses, which may be owing to increased awareness and new targets for general practices through the Investment and Impact Fund (IIF): 2021–2022 and 2022–2023 Structured Medication Reviews SMR-03: percentage of patients prescribed a DOAC who received a renal function test and had their weight and CrCl, along with a change or confirmation of their medication dose.^12^ Overdosing was highest for rivaroxaban, which is in line with data in Supplementary Table S1. We found apixaban was highest for underdosing; however, Khachatryan *et al* found that dabigatran followed by apixaban were highest for underdosing.^11^ A systematic review of 75 studies showed that most patients treated with DOACs for stroke prevention in AF received doses in accordance with guidelines. However, 25%–50% of patients received off-label doses. DOAC overdosing was associated with increased all-cause mortality and worse bleeding events while underdosing was associated with increased cardiovascular hospitalisation, particularly for apixaban, with a nearly five-fold increased risk of stroke.^13^

### Implications for practice

To the best of our knowledge, this is the first time that the prescribed DOAC dose has been compared with the calculated dose for recorded CrCl, to determine whether people may be at increased risk of bleeding or reduced efficacy in stroke prevention from their DOAC in England on this scale, since the COVID-19 pandemic.

In our analysis, 316 947 patients were aged ≥70 years. While this data is in line with an expected increase in DOAC prescribing based on national guidance, extra vigilance in the older age group is suggested given the increasing use of DOACs in older patients and the Medicines and Healthcare products Regulatory Agency (MHRA) warning that DOACs should be used with caution in older patients.^3^

There was a lower percentage of patients with a recorded CrCl compared with eGFR. While National Institute for Health and Care Excellence (NICE) DOAC guidance explicitly mentions CrCl ranges, a commonly used measure of renal function is eGFR.^4,6^ However, use of eGFR for dosing of DOACs is known to increase the risk of bleeding events as a consequence of overestimating renal function.^5^ The MHRA advises that CrCl should be considered for dosage adjustment of medicines that are substantially renally excreted and have a narrow therapeutic index. In particular, CrCl should always be used to guide dose adjustment for DOACs.^5^

With the increasing numbers of patients on DOACs in primary care, it is important to ensure guidelines are explicit that eGFR and CrCl are not the same nor equivalent measures, and that tools exist to make calculation and recording of CrCl easier for healthcare professionals to document in clinical systems to allow for review, audit, and service evaluation. In addition, there is uncertainty whether to use actual or ideal body weight in estimating CrCl.^14^ Evidence suggests the Cockcroft–Gault equation is less accurate in extremes of bodyweight (underweight and overweight or obesity).^15^ In our study, 4741 patients had a body weight >120 kg and 11 822 had a bodyweight <50 kg (Table 2). For these patients, clarity is needed about which weight to use to calculate CrCl for their DOAC dose.

In England, all general practice clinical systems have sophisticated features to automate certain tasks and provide ‘clinical decision support’ such as automated calculators. Automation of CrCl calculation is complicated by the lack of agreement on which weight to use in the Cockcroft–Gault equation. We think there is an opportunity for national bodies concerned with safety (for example, MHRA, NICE, and NHS England) to collectively identify and update guidelines with which weight is appropriate, amend guidelines to explicitly mention that CrCl is not equivalent to eGFR, and work with GP clinical system suppliers, such as TPP, to standardise the calculation and recording of CrCl in the EHR.

All DOACs should be avoided if CrCl is <15 ml/min^4,6^ and in our study 3196 patients had a recorded CrCl of <15 ml/min (Table 2). These patients should be reviewed and potentially switched to warfarin. Although the majority of patients with recorded AF prescribed a DOAC received the recommended dose, according to their CrCl recorded in our analysis, 26 858 patients received an underdose and 6463 received an overdose, according to their recorded CrCl. These people are at higher risk of bleeding and adverse effects owing to their overdosing, or of not being protected from developing a stroke or systemic embolism from underdosing. Patients prescribed DOACs inappropriately for their CrCl should have their DOAC reviewed to improve their anticoagulant management.

## Supplementary Material

Supplementary data

## Figures and Tables

**Table 1:** Direct-acting oral anticoagulant dose based on renal function in patients with atrial fibrillation^1^

DOAC	Recommended dose	Frequency	CrCl (ml/min)	Codelist
Apixaban	5 mg	Twice daily	≥30	OpenCodelists: DOACs
	2.5 mg	Twice daily	15–29	
Dabigatran	110 mg	Twice daily	>50	
	150 mg			
	110 mg	Twice daily	30–50	
	150 mg			
Edoxaban	60 mg	Once daily	>50	
	30 mg	Once daily	15–50	
Rivaroxaban	20 mg	Once daily	≥50	
	15 mg	Once daily	15–49	

CrCl = creatinine clearance. DOAC = direct-acting oral anticoagulant.

**Table 2:** Patient characteristics of DOAC study patients

		July 2022		February 2023	
Characteristic	Category	Total		Total	
		*n*	%	*n*	%
Total patients on a DOAC		422 539	**100.0%**	430 778	**100.0%**
Non-calculable patients		**878**	**0.2%**	**720**	**0.2%**
Total calculable patients		421 661	**99.8%**	430 058	**99.8%**
Sex	Female	189 669	**44.9%**	191 761	**44.5%**
	Male	231 992	**54.9%**	238 297	**55.3%**
Age, years	18–29	1323	**0.3%**	1355	**0.3%**
	30–39	4191	**1.0%**	4246	**1.0%**
	40–49	10 747	**2.5%**	10 819	**2.5%**
	50–59	28 811	**6.8%**	29 696	**6.9%**
	60–69	64 853	**15.3%**	49 193	**11.4%**
	70–79	139 988	**33.1%**	143 070	**33.2%**
	≥80	171 727	**40.6%**	173 877	**40.4%**
Weight	Recorded	270 696	**64.1%**	313 537	**72.8%**
	of which <50 kg	9820	**3.6%**	11 822	**3.8%**
	of which 50–120 kg	258 211	**95.4%**	296 974	**94.7%**
	of which >120 kg	2665	**1.0%**	4741	**1.5%**
eGFR	Recorded	375 685	**88.9%**	398 214	**92.4%**
Serum creatinine level	Recorded	383 535	**90.8%**	406 335	**94.3%**
Creatinine clearance	Recorded	222 897	**52.8%**	316 553	**73.5%**
	of which <15 ml/min	2563	**1.1%**	3196	**1.0%**
Serum creatinine but no creatinine clearance	Recorded	160 638	**38.0%**	89 782	**20.8%**
DOAC indication	AF	313 329	**74.2%**	318 169	**73.9%**
	Other indications	108 332	**25.6%**	111 889	**26.0%**
AF and CrCl	Recorded	174 056	**41.2%**	243 936	**56.7%**
Calculated DOAC dose compared with prescribed dose in AF where CrCl recorded*	Match	146 429	**84.4%**	208 837	**86.2%**
	Over	5585	**3.2%**	6463	**2.7%**
	Under	21 400	**12.3%**	26 858	**11.1%**

AF = atrial fibrillation. CrCl = creatinine clearance. DOAC = direct-acting oral anticoagulant. eGFR = estimated glomerular filtration rate

* Where the prescribed DOAC dose was available.

**Table 3:** Change in recording of weight, eGFR, creatinine, CrCl in patients prescribed a DOAC (July 2022–February 2023)

Parameter recorded	Number (%) of patients with data recorded (July 2022)	Number (%) of patients with data recorded (February 2023)	Change in number (%) of patients (July 2022–February 2023)
Weight	270 696 (64.1)	313 537 (72.8)	+42 841 (+8.7)
eGFR	375 685 (88.9)	398 214 (92.4)	+22 529 (+3.5)
Creatinine	383 535 (90.8)	406 335 (94.3)	+22 800 (+3.5)
CrCl	222 897 (52.8)	316 553 (73.5)	+93 656 (+42.0)
Creatinine and no CrCl	160 638 (38.0)	89 782 (20.8)	–70 856 (–17.2)
AF diagnosis	313 329 (74.2)	318 169 (73.9)	+4840 (+0.3)
AF diagnosis+ CrCl	174 056 (41.2)	243 936 (56.7)	+69 880 (+15.4)

AF = atrial fibrillation. CrCl = creatinine clearance. DOAC = direct-acting oral anticoagulant. eGFR = estimated glomerular filtration rate

## How this fits in

Direct-acting oral anticoagulants (DOACs) require renal function tests and creatinine clearance (CrCl) calculated so safe and effective doses are prescribed, but CrCl is not being recorded in a substantial number of patients. There is no national agreement on which weight to use in CrCl calculations and, where GP clinical systems have internal CrCl calculators, these are inconsistent on which weight is used. With the increasing number of people on DOACs, there is an urgent need to have national agreement on which weight is used in CrCl calculations. National organisations tasked with safety should work with GP clinical systems suppliers to facilitate automated CrCl recording when renal function tests are recorded.

## References

[1] NHS England, NHS Improvement (2020). Clinical guide for the management of anticoagulant services during the coronavirus pandemic.

[2] Afzal S, Zaidi STR, Merchant HA (2021). Prescribing trends of oral anticoagulants in England over the last decade: a focus on new and old drugs and adverse events reporting. J Thromb Thrombolysis.

[3] Medicines and Healthcare products Regulatory Agency (2020). Direct-acting oral anticoagulants (DOACs): reminder of bleeding risk, including availability of reversal agents. Drug Safety Update.

[4] Joint Formulary Committee (2024). British National Formulary.

[5] Medicines and Healthcare products Regulatory Agency (2019). Prescribing medicines in renal impairment using the appropriate estimate of renal function to avoid the risk of adverse drug reactions.

[6] National Institute for Health and Care Excellence (NICE) (2023). Anticoagulation — oral.

[7] Curtis HJ, MacKenna B, Walker AJ (2021). OpenSAFELY: impact of national guidance on switching anticoagulant therapy during COVID-19 pandemic. Open Heart.

[8] Fisher L, Speed V, Curtis HJ (2022). Potentially inappropriate prescribing of DOACs to people with mechanical heart valves: A Federated analysis of 57.9 million patients’ primary care records in situ using OpenSAFELY. Thromb Res.

[9] NICE (2023). Scenario: Edoxaban.

[10] NHS Business Services Authority (2023). Pseudo anonymised patient level prescribed and dispensed data set January 2018 to February 2023.

[11] Khachatryan A, Doobaree IU, Spentzouris G (2023). Direct oral anticoagulant (DOAC) dosing in patients with non-valvular atrial fibrillation (NVAF) in the United kingdom: A retrospective cohort study using CPRD Gold database. Adv Ther.

[12] NHS England (2021). Annex B–Investment and Impact Fund (IIF): 2021/22 and 2022/23.

[13] Santos J, António N, Rocha M, Fortuna A (2020). Impact of direct oral anticoagulant off-label doses on clinical outcomes of atrial fibrillation patients: a systematic review. Br J Clin Pharmacol.

[14] Erskine D (2019). DOAC dosing in renal impairment. Drug Ther Bull.

[15] Cockcroft DW, Gault MH (1976). Prediction of creatinine clearance from serum creatinine. Nephron.

[16] NHS England (2023). The NHS England OpenSAFELY COVID-19 service — privacy notice.

[17] NHS England (2023). Data security and protection toolkit.

[18] NHS England (2022). ISB1523: Anonymisation standard for publishing health and social care data.

[19] Department of Health and Social Care (2022). Coronavirus (COVID-19): notice under regulation 3(4) of the Health Service (Control of Patient Information) Regulations 2002 — general.

[20] NHS England (2020). COVID-19 public health directions 2020.

[21] NHS Health Research Authority (2023). Confidentiality Advisory Group.

