# Supplementary material for: Safety of direct-acting oral anticoagulant (DOAC) prescribing: OpenSAFELY-TPP analysis of 20.5 million adults’ electronic health records
Source: BJGP Open. Author manuscript; Available in PMC 2024 Jul 31. (PMC7616327; doi:10.3399/BJGPO.2023.0163)
Supplement: Supplementary file 1 [file EMS197237-supplement-Supplementary_data.pdf]

Table S1:
Patient Characteristics of DOAC study patients

Jul-22

Characteristic	Category	Apixaban n	Apixaban %	Dabigratan n	Dabigratan %	Edoxaban n	Edoxaban %	Rivaroxaban n	Rivaroxaban %
Total patients on a DOAC									
Non calculable patients									
Total calculable patients		203153	48.2%	9209	2.2%	79679	18.9%	129620	30.7%
Sex	Female	95046	50.1%	3532	1.9%	34877	18.4%	56214	29.6%
	Male	108107	46.6%	5677	2.4%	44802	19.3%	73406	31.6%
Age years)	18-29	626	47.3%	12	0.9%	91	6.9%	594	44.9%
	30-39	1790	42.7%	60	1.4%	335	8.0%	2006	47.9%
	40-49	4632	43.1%	162	1.5%	906	8.4%	5047	47.0%
	50-59	12787	44.4%	496	1.7%	3704	12.9%	11824	41.0%
	60-69	29925	46.1%	1409	2.2%	10917	16.8%	22602	34.9%
	70-79	66070	47.2%	3573	2.6%	27594	19.7%	42751	30.5%
	80+	87323	50.8%	3494	2.0%	36124	21.0%	44786	26.1%
Weight	Recorded	125381	46.3%	5676	2.1%	61899	22.9%	77740	28.7%
	of which <50Kg	5267	53.6%	131	1.3%	2018	20.5%	2404	24.5%
	of which 50-120Kg	118908	46.1%	5494	2.1%	59510	23.0%	74299	28.8%
	of which >120Kg	1206	45.3%	51	1.9%	371	13.9%	1037	38.9%
eGFR	Recorded	180765	48.1%	8249	2.2%	73076	19.5%	113595	30.2%
Serum creatinine level	Recorded	185306	48.3%	8268	2.2%	73529	19.2%	116432	30.4%
Creatinine clearance	Recorded	104885	47.1%	4459	2.0%	47694	21.4%	65859	29.5%
	of which <15mL/min	1263	49.3%	52	2.0%	460	17.9%	788	30.7%
Serum creatinine but no creatinine clearance	Recorded	80421	50.1%	3809	2.4%	25835	16.1%	50573	31.5%
DOAC indication	AF	154598	49.3%	8004	2.6%	69045	22.0%	81682	26.1%
	Other indications	48555	44.8%	1205	1.1%	10634	9.8%	47938	44.3%
AF and CrCl	Recorded	83299	47.9%	3931	2.3%	42156	24.2%	44670	25.7%
Calculated DOAC dose compared to prescribed dose in AF where CrCl recorded	Match	66726	45.6%	2636	1.8%	37598	25.7%	39469	27.0%
	Over	1264	22.6%	252	4.5%	1642	29.4%	2427	43.5%
	Under	14410	67.3%	1943	9.1%	2916	13.6%	2131	10.0%

Feb-23

Characteristic	Category	Apixaban n	Apixaban %	Dabigratan n	Dabigratan %	Edoxaban n	Edoxaban %	Rivaroxaban n	Rivaroxaban %
Total patients on a DOAC									
Non calculable patients									
Total calculable patients		194137	45.1%	7955	1.8%	110541	25.7%	117425	27.3%
Sex	Female	90288	47.1%	3076	1.6%	48006	25.0%	50391	26.3%
	Male	103849	43.6%	4879	2.0%	62535	26.2%	67034	28.1%
Age years)	18-29	660	48.7%	16	1.2%	98	7.2%	581	42.9%
	30-39	1898	44.7%	68	1.6%	344	8.1%	1936	45.6%
	40-49	4751	43.9%	169	1.6%	1126	10.4%	4773	44.1%
	50-59	12991	43.7%	440	1.5%	4757	16.0%	11508	38.8%
	60-69	29383	59.7%	1241	2.5%	15226	31.0%	3343	6.8%
	70-79	62908	44.0%	3051	2.1%	38730	27.1%	38381	26.8%
	80+	81586	46.9%	2963	1.7%	50252	28.9%	39076	22.5%
Weight	Recorded	139762	44.6%	5800	1.8%	85002	27.1%	82973	26.5%
	of which <50Kg	5903	49.9%	128	1.1%	3267	27.6%	2524	21.4%
	of which 50-120Kg	131733	44.4%	5592	1.9%	80976	27.3%	78673	26.5%
	of which >120Kg	2126	44.8%	80	1.7%	759	16.0%	1776	37.5%
eGFR	Recorded	179282	45.0%	7390	1.9%	104622	26.3%	106920	26.8%
Serum creatinine level	Recorded	183348	45.1%	7415	1.8%	105905	26.1%	109667	27.0%
Creatinine clearance	Recorded	140477	44.4%	5560	1.8%	86598	27.4%	83918	26.5%
	of which <15mL/min	1449	45.3%	33	1.0%	560	17.5%	1154	36.1%
Serum creatinine but no creatinine clearance	Recorded	42871	47.8%	1855	2.1%	19307	21.5%	25749	28.7%
DOAC indication	AF	143658	45.2%	6807	2.1%	97716	30.7%	69988	22.0%
	Other indications	50479	45.1%	1148	1.0%	12825	11.5%	47437	42.4%
AF and CrCl	Recorded	107761	44.2%	4810	2.0%	77866	31.9%	53499	21.9%
Calculated DOAC dose compared to prescribed dose in AF where CrCl recorded	Match	87536	41.9%	3287	1.6%	70356	33.7%	47658	22.8%
	Over	1392	21.5%	210	3.2%	2303	35.6%	2558	39.6%
	Under	17806	66.3%	1313	4.9%	5207	19.4%	2532	9.4%

Figure S1

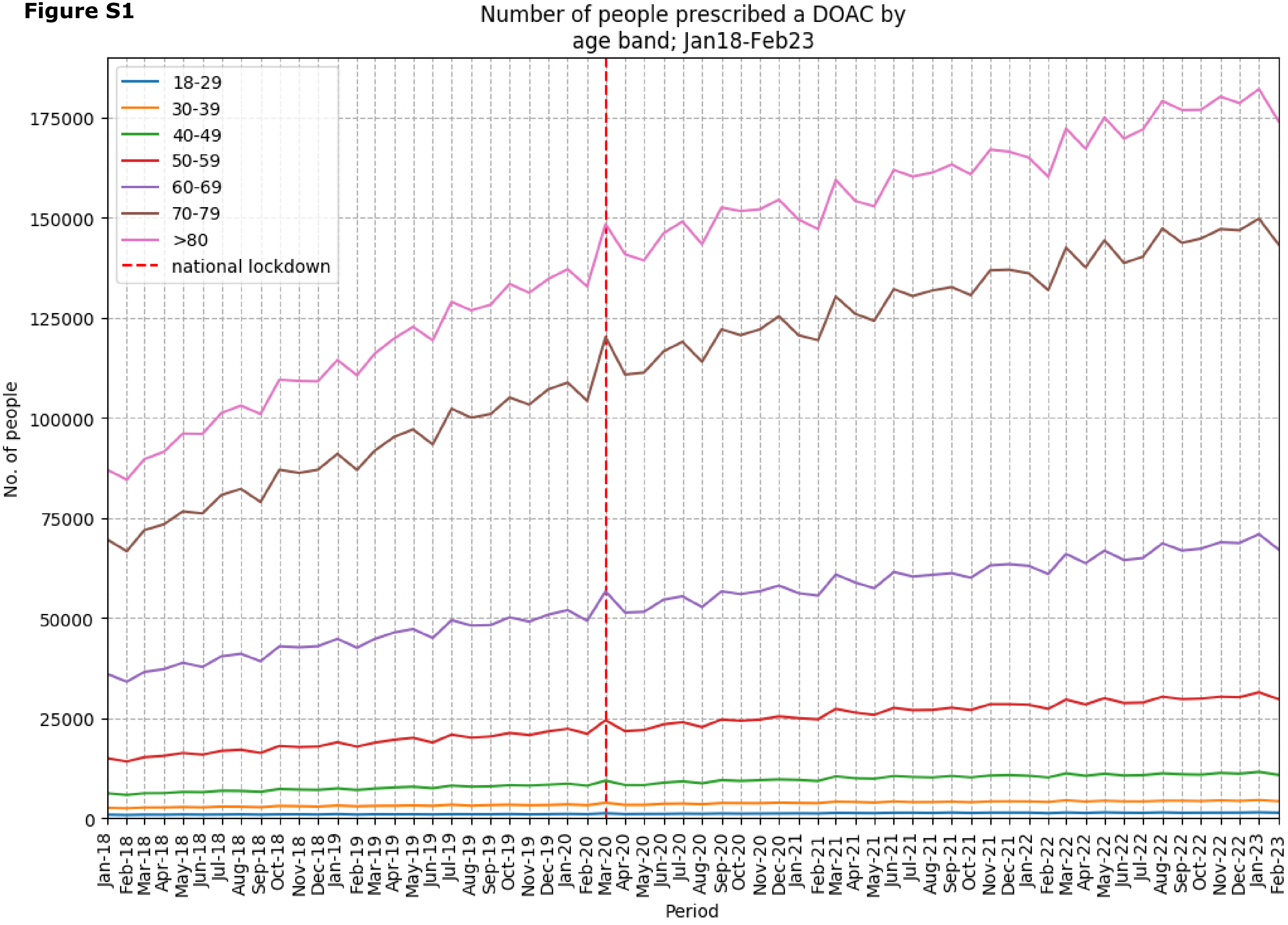
Number of people prescribed a DOAC by age band; Jan18-Feb23
18-29
30-39
40-49
50-59
60-69
70-79
>80
national lockdown
No. of people
0
25000
50000
75000
100000
125000
150000
175000
Period
Jan-18 Feb-18 Mar-18 Apr-18 May-18 Jun-18 Jul-18 Aug-18 Sep-18 Oct-18 Nov-18 Dec-18 Jan-19 Feb-19 Mar-19 Apr-19 May-19 Jun-19 Jul-19 Aug-19 Sep-19 Oct-19 Nov-19 Dec-19 Jan-20 Feb-20 Mar-20 Apr-20 May-20 Jun-20 Jul-20 Aug-20 Sep-20 Oct-20 Nov-20 Dec-20 Jan-21 Feb-21 Mar-21 Apr-21 May-21 Jun-21 Jul-21 Aug-21 Sep-21 Oct-21 Nov-21 Dec-21 Jan-22 Feb-22 Mar-22 Apr-22 May-22 Jun-22 Jul-22 Aug-22 Sep-22 Oct-22 Nov-22 Dec-22 Jan-23 Feb-23

Figure S2

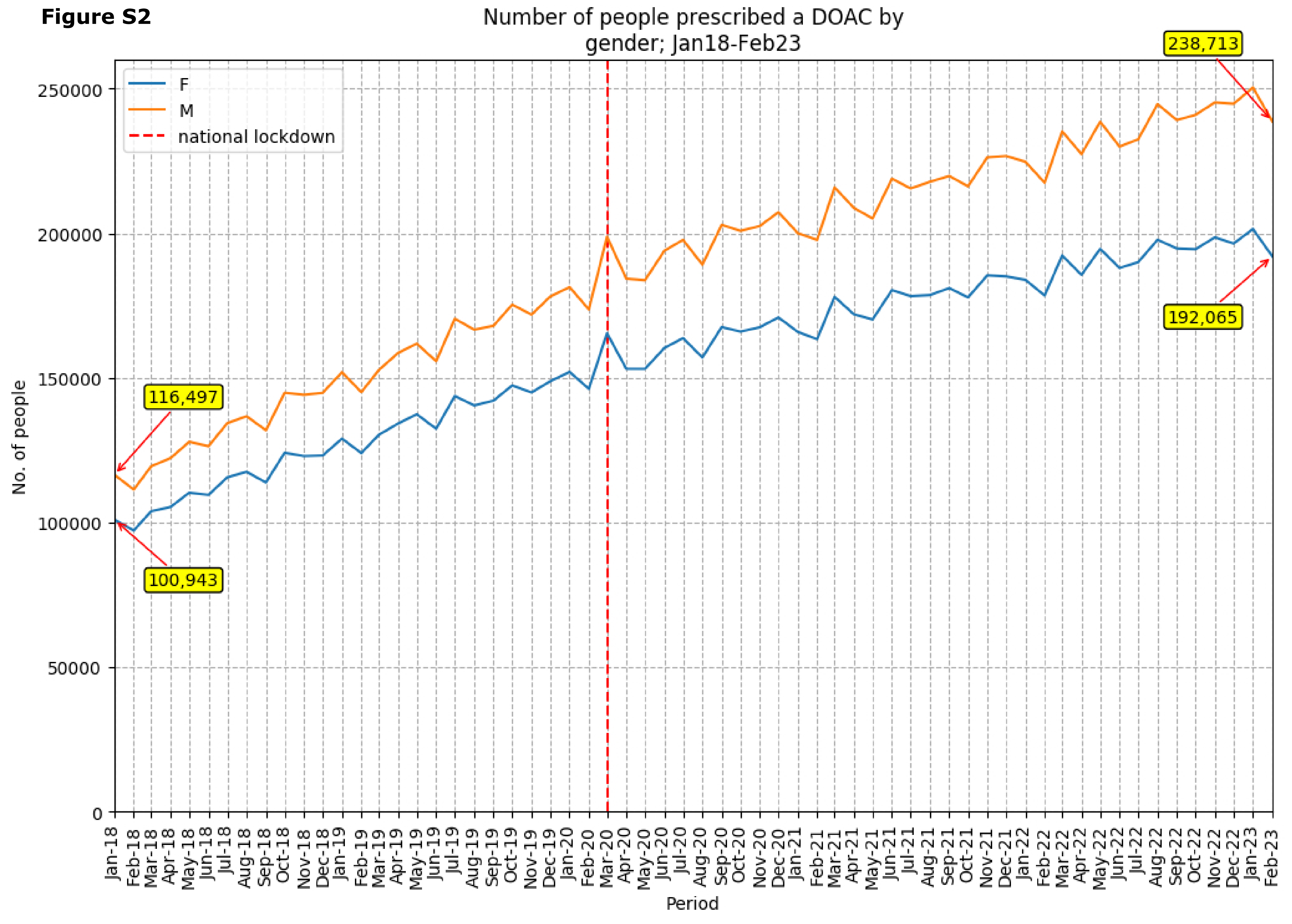

Number of people prescribed a DOAC by gender; Jan18-Feb23
F
M
national lockdown
238,713
192,065
116,497
100,943
250000
200000
150000
100000
50000
0
No. of people
Period
Jan-18
Feb-18
Mar-18
Apr-18
May-18
Jun-18
Jul-18
Aug-18
Sep-18
Oct-18
Nov-18
Dec-18
Jan-19
Feb-19
Mar-19
Apr-19
May-19
Jun-19
Jul-19
Aug-19
Sep-19
Oct-19
Nov-19
Dec-19
Jan-20
Feb-20
Mar-20
Apr-20
May-20
Jun-20
Jul-20
Aug-20
Sep-20
Oct-20
Nov-20
Dec-20
Jan-21
Feb-21
Mar-21
Apr-21
May-21
Jun-21
Jul-21
Aug-21
Sep-21
Oct-21
Nov-21
Dec-21
Jan-22
Feb-22
Mar-22
Apr-22
May-22
Jun-22
Jul-22
Aug-22
Sep-22
Oct-22
Nov-22
Dec-22
Jan-23
Feb-23
